# Symptoms of depression and anxiety in adolescents and their caregivers: A cross-sectional study from North Macedonia

**DOI:** 10.1192/j.eurpsy.2023.473

**Published:** 2023-07-19

**Authors:** I. Kunovski, F. Bolinski, M. Raleva, R. Isjanovska, G. Kalpak, A. Novotni, B. Stefanovski, K. Hadzihamza, S. Bajraktarov

**Affiliations:** ^1^University Clinic of Psychiatry, Medical Faculty, Ss. Cyril and Methodius University, Skopje, North Macedonia; ^2^Netherlands Institute for Mental Health and Addiction - Trimbos Institute, Utrecht, Netherlands; ^3^Institute for Epidemiology and Biostatistics with Medical Informatics, Skopje, North Macedonia

## Abstract

**Introduction:**

Mental health problems have increased internationally during the COVID-19 pandemic. However, most data stems from Western countries and there is a clear lack of prevalence rates and potential risk factors from Central and Eastern Europe.

**Objectives:**

To investigate the point prevalence and to provide further information on risk factors of depressive and anxiety symptoms in adolescents and their caregivers in North Macedonia after the COVID-19 pandemic.

**Methods:**

A cross-sectional survey study was conducted on adolescents and their caregivers through the school setting in different areas of North Macedonia. Survey items assessed symptoms of depression, anxiety, and respondents’ fear of COVID-19, as well as a number of risk factors, such as gender and living environment.

**Results:**

506 adolescents and 492 caregivers completed the survey. Symptoms of depression and anxiety were mild to moderate in adolescents and their caregivers. Women and girls generally scored higher than men and boys, and adolescents in high school scored higher than those in elementary school. Prevalence rates for depression were 29.2% for adolescents and 10.4% for caregivers, while rates of anxiety were 23.7% for adolescents and 6.1% for caregivers.

**Conclusions:**

This study provides a first insight into the mental health of adolescents and their caregivers after the COVID-19 pandemic in North Macedonia. Further research is required to investigate the relatively low reported rates of caregivers’ mental health problems compared to data from other countries.

**Disclosure of Interest:**

None Declared

